# The Institutional Building of Science and Innovation Diplomacy in Latin America: Toward a Comprehensive Analytical Typology

**DOI:** 10.3389/frma.2021.654358

**Published:** 2021-04-27

**Authors:** Renan Gonçalves Leonel da Silva, Gabriela Gomes Coelho Ferreira, Janina Onuki, Amâncio Jorge Nunes de Oliveira

**Affiliations:** ^1^Health Ethics and Policy Lab, Department of Health Sciences and Technology, ETH Zurich, Zurich, Switzerland; ^2^Department of Preventive Medicine, Faculty of Medicine of the University of São Paulo, São Paulo, Brazil; ^3^Center for International Negotiation Studies, Institute of International Relations, University of São Paulo, São Paulo, Brazil

**Keywords:** Science and Innovation Diplomacy, science, innovation, institutional building, interdisciplinary, Latin America

## Abstract

Science and Innovation Diplomacy (S&ID) has emerged in recent years as a relevant scholarly movement and interdisciplinary research agenda internationally. This field is promoting a significant impact on the understanding of the cultural and political dynamics of Science, Technology and Innovation (ST&I), implementing initiatives from local to global level. Notwithstanding, S&ID is growing asymmetrically around the world, setting up over a particular configuration in the so-called Global South (GS) societies. In Latin America (LA), although S&ID is a recent, unequal and intra-nationally fragmented process, there are important achievements that have been able to create a favorable mix of approaches, agendas, and practices in this field. Addressing the scope of the special issue “Science Diplomacy and Sustainable Development: Perspectives from Latin America,” this article aims to present a comprehensive analytical typology to the study of the emerging experiences of S&ID in LA, catching the diversity of this research agenda. This is a qualitative merged method-based study, sustained by a literature review, documentary research, online data analysis, and typology building. We understand S&ID in LA as a tentative re-organization of different states and subnational actors around the study and institutionalization of the governance of contemporary transformations on the systems of ST&I.

## Introduction

Science and Innovation Diplomacy (S&ID) has been significantly impacting the understanding of the cultural and political dynamics of Science, Technology and Innovation (ST&I) over the last few years, from local to global level. We understand S&ID as a relatively recent scholarly movement and interdisciplinary research agenda addressing the study of how state and subnational actors interfere in the supranational governance of ST&I, as well as the idealization and management of tools of tentative governance regarding this field in different cultural settings. A relevant question about S&ID can be how those actors are capable of reconfiguring political and sociotechnical infrastructures to foster broader and more independent regimes of governance of science and innovation in different national contexts, by strengthening tools of international collaboration to sustain the potential of virtually permanent supranational initiatives.

However, there are multiple interpretations of what this field is or could be. As set by the “Madrid Declaration on Science Diplomacy” (S4D4C, 2019), “Science diplomacy (…) is understood as a series of practices at the intersection of science, technology and foreign policy.” Moreover, according to the board of experts who signed this declaration, the contemporary interest in the S&ID debate “comes in response to identified challenges at the interface of science and foreign policy, where a greater scientific voice could both add value to bi- and multilateral discussions and decisions about our shared global concerns.” As the paper shows, defining the concept of S&ID is, itself, an interesting intellectual exercise and admits several analytical dimensions, including behavioral, political–institutional, tentative governance, social/academic movement, and technoscientific networking.

Despite the development of well-known initiatives of S&ID in the European Union (EU), S&ID has been growing asymmetrically around the world. It motivated us to call attention to how this field has been built over a particular configuration in the so-called Global South (GS) societies. In those countries, S&ID shares both similarities and strong asymmetries in terms of its historical and political trajectories, governmental agendas, approaches to Science and Technology Policy (S&TP), regimes of knowledge production, infrastructures, etc.

In Latin America (LA), S&ID is a recent and intra-nationally fragmented process; however, there are important emerging initiatives in the field, stimulating a broad debate about the mix of approaches, agendas, and practices for S&ID. There, the field has been established at universities, research institutes, and non-governmental organizations (NGOs), with a timid presence in the private sector, media, and the broad public opinion (CILAC, 2020). It is presenting itself as a challenge to S&ID in GS, where those institutions should be capable of sensitizing a broader spectrum of stakeholders to achieve sustainable scientific and technological cooperation at the academic, private, and diplomatic sphere and beyond.

Addressing the scope of the special issue “Science Diplomacy and Sustainable Development: Perspectives from Latin America,” this article aims to present an analytical typology to the study of the emerging experiences of S&ID in LA, catching the diversity of this research agenda. This is a qualitative merged method-based study, supported by a literature review, documentary research, online data analysis, and typology building.

Thus, to contribute to this research agenda internationally, this paper brings interdisciplinary theoretical and methodological approaches, suggesting that the understanding of the field of S&ID in LA can greatly benefit from a broader review of literature, and that the specificity of the regional cases can be discussed in the analytical typology proposed here.

## Methods

The paper presents the results of a qualitative-based merged method study about the emergence of initiatives and policies of S&ID in LA in different national contexts. We supported our analysis with a combination of four strategies and data collection: a literature review, documentary research, online data analysis, and the building of an analytical typology based on selected cases of institutional building in S&ID in LA. All the data collection is based on the scholarly experience of the authors in the field, as well as their involvement within two sessions of the so-called “São Paulo School of Advanced Sciences on Science and Innovation Diplomacy” (InnSciD), formerly sponsored by the São Paulo Research Foundation (FAPESP), which took place in 2019 at the campus of the University of São Paulo in the city of São Paulo, Brazil, and virtually in 2020. More details and explanation of the methodological steps are described below.

### Empirical Research Design

Empirical research design (ERD) is a research strategy that improves the methodological and analytical framework using evidence of experiences and empirical validation of relevant cases (Mills et al., 2010). Due to the coronavirus disease 2019 (Covid-19) outbreak, the ERD for this paper took place virtually in Brazil, and the collection of primary and secondary data was conducted between February and June 2020. During this period, the authors were promoting webinars and e-symposiums as part of the activities of the Centre for International Negotiation Studies of the University of São Paulo's Institute of International Relations, where several PhD students, researchers, and guest speakers presented developments of their investigation and interesting emerging debates related to the field of S&ID.

### Data Collection

Primary and secondary data were collected through a literature review and the documentary research, mainly in online sources, which highlighted the importance of recent initiatives of S&ID at local level and a growing agenda of international collaboration between groups in LA and EU (Sánchez, 2018). Moreover, material was selected from important non-state actors in academia, national authorities, professionals from business sectors, and people from NGOs involved in the production of reports, discussion papers, and presentations about the initiatives of S&ID in conferences and professional events internationally, in person in 2019 and virtually in 2020.

Despite focusing on the description of some patterns of the experiences in S&ID in LA, an interesting achievement of this article is the critical analysis about the role of foreign policy and its tools in bridging the gap more broadly between science and technology policies and society. Hence, the emergence of S&ID can be a response to the failure of state actors to promote better governance to solve the lack of competitiveness of knowledge-based sectors in the region. The data collection also feeds the interdisciplinary debate presented in this article on how different literatures can help to understand the incapability of governments to create and maintain a more efficient and sustainable agenda for ST&I in LA.

According to the review, all selected cases were reported in articles and editorials regarding the experiences of S&ID. Documents were scrutinized that describe emerging relevant initiatives under design and implementation in different national contexts of LA. In addition, a documentary research was conducted in the available files of the two sessions of the InnSciD conferences in Sao-Paulo, Brazil. There, the following were found: 12 presentations of the main invited speakers, one official report of the conference, and eight abstracts of the current research of PhD students, postdoctoral researchers, guest scholars, and other partners from NGOs, academia, EU, and other international boards. The case selection is described in the section below.

### Case Selection

Reviewing articles and reports and conducting documentary research in the files of the InnSciD have shown a variety of experiences of S&ID in LA. To organize the case selection, firstly, relevant actors and institutions were separated into two categories: state and non-state actors. State actors are understood here as only national cases in which the structure for S&ID is under the umbrella of the foreign policy at the national/federal level, or other initiatives exclusively centralized and managed by governmental authorities, its professionals, and internal competencies. Regarding the non-state actors, these are institutions that merge different *rationales* from other sectors of society, mainly academic-oriented groups, NGOs, and initiatives between the public and private sectors, which can involve the state, but not as a coordinator or central managers of the institutional building and development.

Finally, a number of 21 actors of institutional building in LA were selected, composed of: 12 state actors/countries (i.e., Brazil, Mexico, Bolivia, Uruguay, Cuba, Panama, Paraguay, Chile, Colombia, Costa Rica, Guatemala, and Organization of Ibero-American States OEI) and 9 non-state actors (i.e., Inter-American Network of Academy of Sciences IANAS, Latin American Academy of Sciences ACAL, Open Forum of Science in Latin America and Caribe CILAC, Bolivian Observatory of Science BOS, Uruguayan Technological Consulate in San Francisco UTCSF, Regional Leaders Summit RLS, São Paulo School of Advanced Sciences on Science and Innovation Diplomacy InnSciD, Porto Digital of Recife, and the Cuban Academy of Sciences CAS).

### Coding

The main trends in S&ID in the 21 selected actors and institutions were cataloged and positioned in six different analytical categories, regarding the way they are structured in terms of their main goals and sphere of influence and governance. Finally, Table 1 was created with a concise description of the relevant policies and initiatives promoted by those institutions in those countries. The data are collected and presented in Table 2.

**Table 1:** Categories of the analytical typology (*N* = 6).

	**Group**	**Type of initiative**
Non-Institutionalized initiatives	A	*ad-hoc* summits and expert panels
Under institutionalization	B	Academic-oriented projects
Institutionalized initiatives	C	Observatories and tentative regimes
	D	Agendas of non-governmental organizations
	E	Tools of Foreign Policy
	F	International organizations and supranational programs

**Table 2:** Distribution of selected actors and institutions in the typology (*N* = 21).

**Group**	**Name**	**Composed mainly by**	**Institution**	**About**	**Is it a state or non-state driven initiative?**
A	Non-institutionalized initiatives	*ad-hoc* summits and expert panels	Cuban Academy of Sciences - CAS	The Academy of Sciences of Cuba is an official institution of the Cuban state, national, independent and consultative nature in science, continuing the Royal Academy of Medical, Physical and Natural Sciences of Havana, founded on May 19, 1861, attached to the Ministry of Science, Technology and Environment.	State
			Universidad de los Lannos in Colombia	Program in Science Diplomacy. Among the actions, started a work of identification and motivation of mutual interest between Colombian scientific diaspora to increase cooperation activities, programs and collaborative projects.	Non-state
			Institute of Foreign Service Manuel María Peralta, Puerto Rico.	Ministry of Foreign Affairs through its Institute of Foreign Service Manuel María de Peralta convened a group of notable experts in scientific and technological areas for a discussion about the relations between diplomacy and Science.	
B	Under institutionalization	Academic-oriented projects	Regional Leaders Network – RLS Network	The members of the Regional Leaders Summit are seven key regions across the world, and their strengths extend to science. There are areas where the seven members share existing competencies and excellence in science and innovation.	State/Non-state
			Open Forum of Sciences of Latin America and the Caribbean - CILAC	Open Forum of Sciences of Latin America and the Caribbean (CILAC) is an academic space for debate and exchange about science, innovation and technology.	Non-state
			Sao-Paulo School of Advanced Sciences on Science Diplomacy and Innovation Diplomacy - InnScID SP	InnSciD SP focuses on the academic and professional training of researchers, diplomats and company representatives (InvestSP, 2020) while fostering a rich network of professionals from multidisciplinary backgrounds on S&ID. Since its first edition, InnSciD SP has been evolving into a research program on the subject.	
C	Institutionalized initiatives	Observatories and tentative regimes	Bolivian Observatory of Science BOS - Extraordinary Representative on a Special Mission for Science, Technology and Innovation	Extraordinary Representative on a Special Mission for Science, Technology and Innovation with international organizations and entities in the Silicon Valley, to create the BOS.	State/Non-state
			Uruguayan Technological Consulate in San Francisco	Technological Consulate in San Francisco (USA) for the management of capacities for a better insertion on the global scenario, with the objective of creating opportunities for the national innovative ecosystem	
			Porto Digital of Recife, Brazil	The Porto Digital (Porto Digital, 2021) is a technology park and innovation organization in Brazil, currently working in the fields of Information Technology and Communication, Creative Industries and Urban Technologies. There are representatives of different boards of Ministry of Foreign Affairs, consulates, state/federal stakeholders, and other international partners.	
D	Institutionalized initiatives	Non-governmental Organizations' agendas	The Inter-American Network of Academies of Sciences - IANAS	IANAS is a regional network of Academies of Sciences and it was created with the mission of supporting cooperation to strengthen science and technology as a tool for development in the Americas	Non-state
			Latin America Academy of Sciences - ACAL	ACAL is one of the Academies of Sciences part of IANAS. By focusing on mathematical, physical, chemical, life, and earth sciences, it also intends to increase science cooperation in Latin America and the Caribbean	State/Non-state
E	Institutionalized initiatives	Tools of Foreign Policy	The Foreign Affairs Services, Paraguay	The Foreign Affairs Services of Paraguay declared that S&ID was incorporated into the services in order to potentialize the work of ambassadors.	State
			Diplomatic Academy of Chile Andrés Bello - ACADE	To this end, the Diplomatic Academy of Chile Andrés Bello (ACADE) created the course “Science Diplomacy Formation.”	
			Science Diplomacy Strategy of Panama	Science Diplomacy Strategy created by the Ministry of Foreign Affairs of Panama and the National Secretariat of Science, Technology and Innovation	
			Innovation Diplomacy Program, Brazil	Innovation Diplomacy program deployed by the Ministry of Foreign Affairs through SECTECs, with support of the Apex-Brazil and EMBRAPII	State/Non-state
			Mexican Agency for International Development Cooperation - AMEXCID	Mexican Agency for International Development Cooperation (AMEXCID) has a partnership with the Mexican Ministry of Foreign Relations (SRE) in topics related to S&ID.	
F	Institutionalized initiatives	International Organizations and supranational programs	Organization of Ibero-American states OEI: CTS	Ibero-American Observatory of Science, Technology and Society (CTS) has the objective of strengthening the institutions of Higher Education, where the Ibero-American scientific production is mainly generated.	
			Inter-American Institute for Global Research IAI: STeP	Inter-American Institute for Global Research (IAI) is an intergovernmental body created toward S&ID, creating the IAI Science Technology & Policy (STeP) Fellowship aiming at professional development on three initial pillars of Science Diplomacy, Communication, and Leadership.	State
			Network of Science and Technology Indicators in Ibero-America - RICYT	RICYT was adopted by the CYTED Program as an Ibero-American network and by the Organization of American States (OAS) as an Inter-American network. Today, its main support is the Ibero-American States Organization (OEI), through the Observatory for Science, Technology and Society CTS	
			Ibero-American Science and Technology for Development Program - CYTED	CYTED promotes cooperation in science, technology and innovation for the development of the Ibero-American countries	
			Organization of American States OAS: COMCyT	Inter-American Committee of Science and Technology (COMCyT). Its role is to contribute to the definition and execution of OAS policy on scientific, technological and innovative partnership for development	

### Typology

A wide range of initiatives of S&ID in LA were envisaged regarding the level of institutionalization of the actors and policies involved. The analysis of the institutional building in LA brings a great diversity of experiences, which can be characterized as heterogenous, and in some cases spontaneous and working in a quasi-multi-level dimension, i.e., covering different levels of the state and non-state regimes of governance.

Building a typology was a challenge, since it is not simple to organize such a complex and dynamic number of experiences in the continental level. In the typology, only those experiences in LA were selected that are focused on science diplomacy and innovation diplomacy, strictly considered. There has been excluded from the analysis the signature of agreements encompassing science and technology not yet translated into institutions, programs, courses, departments, or strategies on S&ID.

Furthermore, the typology aims to provide some introductory parameters that can be helpful to analysts to move forward with deeper and more specific studies of those experiences. The main adopted parameter is what the authors call “level of institutionalization,” i.e., how far policies or initiatives went in terms of producing stable agendas of action and in creating routinization of those initiatives over the past few years; moreover, how successful it was to promote stable interconnections with other partners and policies in a multi-level and multi-sectoral perspective (from state actors to subnational entities or from the official Foreign Policy tools to the business sector, academia, or NGOs coordinated initiatives). It was built based on other selected dimensions that drive the institutional building of S&ID in the LA, from non-institutionalized subnational experiences to institutionalized actions at the national level, international collaboration, and state-driven regimes of governance.

Finally, the cases were positioned in the typology based on our understanding about the literature review on S&ID, presentations in the InnSciD SP, and author's experience on the field regarding to how the cases reflect (1) level of impact of scholarly and professional knowledge produced by actors in different national and subnational contexts, (2) level of openness and transparency of the initiatives, (3) level of participation in international collaboration, (4) level of maturity of the S&ID initiatives, and (5) reach of governance between actors in different levels (5). To elaborate this, official data were collected from the review of literature, governmental agencies (as Foreign Ministry's advisory boards, Secretaries of Science and Technology, academic Departments of International Relations, S&T agencies as CONICET, CNPq, CONCYTEC, etc.), other qualified information available online, or shared in the presentation and documents of the InnSciD SP 2019 and 2020. Additionally, data were gathered from reports from the EU and other international boards. A detailed explanation of the limitations of this typology is provided in the Limitations section.

## Results

### S&ID in LA: A Field Under Construction

Mainly coordinated by official state-level actors and institutions after World War II, international collaboration in science and technology is a cultural and political phenomenon in Western societies and has been studied by different fields of humanities and social sciences over the last century. The development of the so-called S&TP occurred in parallel with the emergence of new tools to rationalize the scientific knowledge production in democratic environments. The publication of the report “Science the Endless Frontier,” written by an engineer, Vannevar Bush in 1945 and resulting in the creation of the National Science Foundation in the USA in 1950, can be considered a milestone in this process (Bush, 1945).

At the international level, LA's foreign policy played an important role in international agreements on science and technology regionally. Since the 1920's, they have a long trajectory of S&ID embedded in cultural agreements, and the so-called “cultural diplomacy” explicitly encompassed science and education as the subject of international politics. Furthermore, those agreements aimed at fostering LA countries' soft power abroad and regional integration in the search for development and economic autonomy from the major global powers (Santos, 2009; Ferreira and Oliveira, 2020).

However, we are facing an unprecedent change in the configuration of the policies for ST&I since the late twentieth century, which has been mobilizing non-state actors and civil society. It has also promoted a movement of rethinking about who should drive the translation of scientific knowledge to broader society, and what kind of political framework is better prepared to achieve this sustainability more efficiently, financially, and environmentally. These groups are now building strong geographical decentralized networking and re-designing new regimes of governance of knowledge-based enterprises, that is challenging either governments, industry, and academia at different levels of political and cultural analysis.

S&ID can be considered a result of these macro-economic transformations in the dynamics of States and ST&I, and that is why this is an ongoing process. Selleslaghs (2017) provides a description of the concept of science diplomacy as “a multi-faceted concept” focusing on “diplomacy for science in the meaning of using diplomacy or foreign policy tools to establish stronger cooperation and interaction in the area of research, innovation and higher education, which would eventually benefit one's own research, higher education and innovation capacities” (p. 3).

Leijten (2019) suggests that S&ID is a concept “still under construction,” drawing attention to the fact that “innovation policies are usually closely linked to or embedded in foreign economic policy and trade policies,” i.e., the private sector, and that S&ID depends on a complex interplay between economics, technology, and institutions (p. 2, 3). Likewise, the “São Paulo Framework of Innovation Diplomacy” (2019) defines the field as the set of ideas, strategies, and practices that “lies at the intersection of innovation and foreign policy,” displayed by national and subnational actors, employing diplomatic processes to enhance innovation capabilities. Finally, another useful definition for S&ID is proposed by Aukes et al. (2019), that it would be characterized as a meta-governance approach itself, understood as a constellation of governance arrangements, stakeholders, and *de facto* governance practices. Then, it is an open concept that allows a diversity of approaches, being a dynamic and plastic idea.

However, S&ID did not grow symmetrically around the world. Despite the globalization discourse suggesting a “global wave” of new scientific and technological innovations in the early 2000, affecting all countries in a similar way, LA has shown that it was far from being considered a reality. The continent is historically positioned in the periphery of this global movement and significantly impacts the debate on S&ID since there it is characterized by particular fragmented regimes of knowledge production and different levels of economic performance.

The reasons for the peripheral positioning of LA in the global chains of ST&I are extremely diversified in the literature of Science, Technology and Society (STS) and have been studied by important scholars in the twentieth century, such as Amilcar Herrera and Oscar Vildavisky who delivered great scholarly achievements to Latin American thought on S&T. Recently, Hebe Vessuri, Lea Velho, and Pablo Kreimer (Kreimer, 2019) have presented important contributions about the production, use, and circulation of knowledge in LA as the object of sociological inquiry, as well as its manifestations in politics and culture. However, there is a lack of dialogue between this literature and international relations, diplomacy, geopolitics, and strategy. Hence, this paper can be considered a first step for more investigation in this direction.

There are some possible reasons for the recent development of S&ID in LA^1^, in which can be highlighted: a historical strengthening of national and subnational actors in its capability to interfere in systems of knowledge production and in the diffusion of technologies and innovations beyond the traditional grasp of the official policies for ST&I, i.e., S&TP; the growth of a new sociotechnical infrastructure that has enabled scientists and investors of technology-based business to communicate more efficiently and permanently through the internet and other digital devices, and the geographical complexity of the dynamics of science and technology itself nowadays, that flows at an unprecedented speed. As other international experiences of this field, S&ID in LA can be understood as a coordinated approach and practices between different state and subnational actors around the comprehension of the governance of the global systems of ST&I. As new actors and stakeholders appear, “new infrastructure, competencies and capabilities are required as well as new governance models” (Sánchez, 2018).

### A Typology for the Institutional Building of S&ID in LA

This article proposes a typology to the study of the emerging experiences on S&ID in LA, catching the diversity of this research agenda. The main objective is to identify these experiences in a bi-dimensional basis, taking into account their level of institutionalization and the nature of the main actors involved in its current governance.

#### Non-institutionalized Initiatives: *ad-hoc* Summits and Expert Panels

Non-institutionalized initiatives is the term used here for those that do not focus directly on S&ID itself, even though they do practices that can be fitted in the concept. This category is mainly composed of *ad-hoc* summits and expert panels on S&ID, as they have been developed by Costa Rica, Colombia, and Cuba.

Costa Rica has been promoting strategic thinking and strengthening the capacities of Costa Rican diplomats about the interplay between science and foreign policy. In August 2019, the country convened a group of notable experts in scientific and technological areas for a discussion about the relations between diplomacy and science. The effort was carried out by the Ministry of Foreign Affairs through its “Institute of Foreign Service Manuel María de Peralta” in an attempt to promote S&ID-oriented initiatives (Costa Rica, 2019).

In Colombia, the Ministry of Foreign Affairs presented a document with principles and guidelines for Colombian foreign policy to the years 2018–2022 (Colombia, 2018). The text mentions the use of diplomatic actions for Colombia to be a reference in science, education, and culture. However, science diplomacy is not addressed as a concept. Immersed in the framework of the Foreign Affairs Ministry and the Administrative Department of Science, Technology and Innovation (Colciencias), the University of Llanos started a Program in Science Diplomacy to create a network of the Colombian scientific diaspora. The aim is to increase cooperation activities, programs, and collaborative projects, in the integral development of the region (Unillaños, 2020).

On the occasion of the diplomatic opening between Cuba and the USA in 2015, Cuba's Academy of Sciences CAS and the American Association for the Advancement of Science (AAAS) played an important role paving the road for new collaborations in ST&I between both countries (Jorge-Pastrana et al., 2018). It is also noteworthy that Cuba has a long trajectory in deploying cultural diplomacy on health systems-related topics with international partners, i.e., sending physicians abroad, which can be considered a way to exchange useful knowledge around the world.

#### Initiatives Under Institutionalization: Academic-Oriented Projects

The “initiatives under institutionalization” are characterized by being frequently focused on academic-oriented projects toward science diplomacy and innovation diplomacy. They often not only discuss S&ID from academic grounds but also execute recommendation policy reports and memoranda about the global dynamics of ST&I addressed to inform policymaking in governments and in the private sector. Hence, three initiatives were positioned in this category: The Open Forum of Sciences of Latin America and the Caribbean (CILAC), the São Paulo School of Advanced Sciences on Innovation and Science Diplomacy (InnSciD SP), and The Regional Leaders Summit (RLS-Sciences).

CILAC is an itinerant-based academic space for discussion and exchange of knowledge about the global dynamics of scientific and technological routes. The forum is subscribed by the United Nations Educational, Scientific and Cultural Organization (UNESCO), and every 2 years, the forum promotes a face-to-face meeting addressing the debate on how to strengthen and implement effective initiatives of ST&I in line with the sustainable development goals of the Agenda 2030 (CILAC, 2020).

InnSciD SP started in 2019 as a 2-week summer school event funded by the São Paulo Research Foundation FAPESP and organized by the University of São Paulo's Institute of International Relations, with the support of the Brazilian Ministry of Foreign Affairs. One of the results of the event was the so-called São Paulo Framework Innovation Diplomacy, in which participants included their perspectives and future orientations to the field of S&ID (InnSciD SP, 2019). The event's focus is the academic and professional training of researchers, diplomats, and people from company representatives, aiming at fostering a multidisciplinary networking of researchers and professionals. In 2020, the second edition of the event was online and involved more than 15 speakers from international centers on S&ID, as well as more than 100 participants from 10 different countries, becoming a well-known experience on S&ID in LA.

Lastly, RLS-Sciences is a scientific and research network operating through a multilateral political forum of seven partner regions: Bavaria, Germany; Georgia, USA; Québec, Canada; São Paulo, Brazil; Shandong, China; Upper Austria, Austria; and Western Cape, South Africa. RLS-Sciences is designed to create a sustainable and effective framework for cooperation in ST&I between the seven regions. Even though it is not a Latin American initiative, it involves the region of São Paulo as an active member. The summit's governance consists of three levels of coordinators in each region: Political Coordinators, Scientific Coordinators, and Administrative Coordinators. These boards are partners in multilateral projects and are integrated in scientific networks between and within the regions (RLS-Network, 2020).

#### Institutionalized Initiatives: Observatories and Tentative Regimes

Institutionalized initiatives on S&ID in LA are divided into four groups: “observatories and tentative regimes,” “agendas of non-governmental organizations,” “tools of Foreign Policy,” and “international organizations and supranational programs,” i.e., from less to more institutionalized. Observatories and tentative regimes are more institutionalized initiatives than A and B and are innovating the landscape of the field by their focus on innovation diplomacy and, consequently, wider integration and dialogue with the private sector into their model. Three initiatives were selected in this level: the so-called Porto Digital of Recife-PE, Brazil, The Bolivian Observatory of Science BOS, and the Uruguayan Technological Consulate in San Francisco.

The Porto Digital is a deliberated public policy, created to insert Pernambuco in the technological and innovative scenario of the world. The state government funded a significant amount to implement its infrastructure, and the management of the initiative was implemented through a non-profit civil association, qualified as a Social Organization (OS) by the Government of Pernambuco and by the City of Recife (PCR): the Porto Digital Management Centre (NGPD). The activities of Porto Digital are based on the Triple Helix Model and bring together two business incubators, two business accelerators, two research institutions, a superior education institution in ITC, and several government agencies (Porto Digital, 2021).

In a slightly different model from the current endeavors of LA countries, in 2020, the Government of Bolivia created the position of Extraordinary Representative on a Special Mission for Science, Technology and Innovation (Bolivia, 2020). Curiously, one of the representative's roles is to be based in the Silicon Valley area to facilitate the exchange between researchers, entrepreneurs, and managers for the creation of the “Bolivian Observatory of Science” and the National Fund for Science, Technology and Innovation. The model is similar to that inaugurated by Denmark, the first country to detach a Tech Ambassador to the Silicon Valley and, in some way, puts corporations on the same level as sovereign governments. As expected, it is becoming the object of critics and concern between members of traditional Foreign Policy (Feertchak, 2017).

The same movement has been followed by Uruguay: the country just opened its first Technological Consulate in San Francisco, USA, to be able to promote a better integration of Uruguayan stakeholders in the global ST&I flows, aiming to create opportunities for the National System of Innovation. It is focused on two main goals: linking technology-based companies, universities, and venture capital funds and promoting cooperation with business and local authorities in the USA and Uruguay. This new Uruguayan headquarters in San Francisco will serve as a pilot experience for the implementation of a “Diplomacy in the Digital Era” or “TechPlomacy” (Uruguay, 2020).

#### Institutionalized Initiatives: Agendas of NGOs

As institutionalized initiatives, there are NGOs that have designed specific agendas on S&ID, aiming at increasing scientific cooperation in respective regions and creating a network to guide scientific advice to *policy makers*.

Two regional NGOs stand out in this venture- the Inter-American Network of Academies of Sciences (IANAS) and the Latin America Academy of Sciences (ACAL). IANAS is a regional network of Academies of Sciences, and it was created with the mission of supporting cooperation to strengthen science and technology as agendas for development in the Americas (IANAS, 2020). ACAL is one of the members of the IANAS. By focusing on Mathematics, Physics, Chemistry and Life and Earth Sciences, it also intends to increase science cooperation in the Latin American and the Caribbean regions by creating a net to guide scientific advice to *policy makers*. Its main activities are the development of cooperation programs, including the dissemination of regional scientific events and the evaluation of the research potential of the region with the support of the formation of regional research networks (ACAL, 2020).

#### Institutionalized Initiatives: Tools of Foreign Policy

This category encompasses what we called tools of foreign policy: the initiatives for science and innovation in the official boards of diplomats, deployed by Foreign Affairs Ministries in partnership with other ministries and agencies related to science, education and technology. Here, cases were selected from Brazil, Mexico, Chile, and Panama (Panama, 2019).

Through the Ministry of Foreign Affairs, the Brazilian has a well-established public diplomacy mainly divided into two major areas: “Culture,” “Education,” and “Science, Technology and Innovation,” with a special program focused on innovation diplomacy, the Innovation Diplomacy Program (PDI). The PDI aims at raising the profile of Brazil in relation to foreign innovation ecosystems through activities deployed by Brazilian embassies. These activities are designed around four main targets: (i) identifying partnerships and attracting investments, (ii) supporting the internationalization of Brazilian start-ups, (iii) helping to mobilize diaspora Brazilian scientific research abroad, and (iv) fostering collaboration between Brazilian and foreign technology parks and innovation environments. It is also noteworthy that the Brazilian Foreign Ministry has 54 sectors specialized in ST&I (SECTECs) in its posts abroad (embassies and consulates), in addition to the regional representative offices of the Ministry of Foreign Affairs in several Brazilian capitals. These SECTECs work to explore opportunities for cooperation and project the potential of the Brazilian system of ST&I (Brasil, 2020).

To deploy the PDI strategies, the Brazilian MRE counts on two agencies: Apex-Brasil and Embrapii—The Brazilian Trade and Investment Promotion Agency (Apex-Brasil) to promote Brazilian products and services abroad and to attract foreign investment in strategic sectors of the Brazilian economy. The Brazilian Company of Research and Industrial Innovation (EMBRAPII) is a social organization by the federal public power, which, since 2013, supports technological research institutions fostering the Brazilian industry innovation. The agency operates through intense cooperation with scientific and technological research institutions, public or private, focusing on business demands, and targeting risk sharing in the pre-competitive phase of innovation. The institution maintains a wide policy for international partnerships, with the development of PD&I for the Brazilian industry with foreign companies: to promote the internationalization of companies and advance or share knowledge between countries by means of industrial innovation.

In Mexico, S&ID became important defined targets for the federal government^2^. At the federal level, the Mexican Secretariat for External Relations (SRE) acts to unfold S&ID activities through the Institute of Mexicans Abroad (IME), the Mexican International Cooperation Agency for Development (AMEXCID), and the National Council of Science and Technology (CONACYT). On the one hand, AMEXCID is a decentralized body of the Ministry of Foreign Relations (SRE) to address issues related to International Cooperation for Development, including educational, cultural, technical and scientific, and economic efforts (Ciudad De Mexico, 2020). On the other hand, the SRE and the CONACYT created the SRE-CONACYT sector research fund to the strengthening of scientific capacities and the diffusion in the areas of knowledge that the SRE requires. Finally, the IME works on creating a strategic network of Mexicans abroad to manage the brain circulation.

Science diplomacy has become a strategic objective in the training of future Chilean and Paraguayan diplomats. While the Diplomatic Academy of Chile Andrés Bello (ACADE) created, in 2019, the course “Science Diplomacy Formation” (Chile, 2019), the Foreign Affairs Services of Paraguay declared in May 2020 that S&ID was incorporated into the services in order to potentialize the work of ambassadors, as one of the consequences of the Covid-19 pandemic (Paraguay, 2020).

In August 2018, Panamá launched the Panamanian National Strategy on Science, Technology and Innovation. The document was created to identify simple actions in the short and medium terms to promote science diplomacy as the interaction between leaders and regional experts, on the verge of science and politics. The main goal is to use science diplomacy to meet local, regional, and global challenges. The National Strategy was created by the Foreign Affairs Ministry (MIREX) and the National Secretariat of Science, Technology and Innovation (SENACYT), with the support of the UNESCO, the American Association for the Advancement of Science (AAAS), and the Spanish Foundation for Science and Technology (FECYT).

The Panamanian strategy was built around three pillars that stem from the framework created by the Royal Society and the AAAS in 2009: science for diplomacy, diplomacy for science, and science in diplomacy. Each pillar has objectives and suggested actions to achieve the better management of science diplomacy as a tool for development and for the solution of global problems that have impacted on national societies.

#### Institutionalized Initiatives: International Organizations and Supranational Programs

Multiple actors and different levels of international governance are crucial to the development of efficient S&ID initiatives. International organizations are important to foster S&ID and, in LA, among the intergovernmental endeavors, there are significant agendas being built over the last few decades. International organizations have played an important role in creating networks, committees, and programs within their context to account for higher education, science, and innovation, and in this category, the Ibero-American Science and Technology for Development Program (CYTED), the Organization of Ibero-American States (OEI), the Organization of American States (OAS), and the Inter-American Institute for Global Research (IAI) were selected.

An important example of this is the Ibero-American Science and Technology for Development Program (CYTED). It was created in the early 1980's by the governments of Ibero-American countries to promote cooperation in ST&I within those countries. CYTED works through different financing instruments that mobilize Ibero-American entrepreneurs, researchers, and specialists addressing the development of science and innovation projects. With a dual structure that combines institutional and functional bodies, the program has signatory agencies of participant countries, commonly responsible for their Science Policy and their relationship with governments (Gual-Soler, 2014).

Similarly, OEI is another relevant example of institutional building in S&ID, which has been establishing important programs on science, technology, and education. One of them, the Ibero-American Observatory of Science, Technology and Society (CTS), has the objective of strengthening the institutions of higher education, where the Ibero-American scientific production is mainly generated. The observatory is aimed at obtaining evidence on the capabilities, challenges, and opportunities of the Ibero-American countries in the field of science and technology, as well as on their aptitudes for the practice of scientific research, or technological development and innovation.

Among its main activities, the Network of Science and Technology Indicators in Ibero-America (RICYT) has been implemented since 1995. Recently, RICYT was adopted by the CYTED Program as an Ibero-American network and by the Organization of American States (OAS) as an Inter-American network (RICyT, 2020). It can be affirmed that those initiatives compose a group of what we call the “Ibero-American agenda for S&ID” since the level of interconnected actions, international governance, and cultural similarities are making possible great achievements in this field.

In the hemispheric dimension of the Americas, the OAS also dived into the subject of S&ID by creating the Inter-American Committee of Science and Technology (COMCyT). Its role is to contribute to the definition and execution of OAS policy on scientific, technological, and innovative partnership for development by coordinating activities on science and technology internationally. The committee had its first meeting in 1998 and, according to the OAS website, held the last regular meeting in 2013. In November 2017, the COMCyT authorities were elected during the fifth meeting of ministers and high authorities on science and technology (V REMCYT) in Medellin, Colombia (OAS, 2021). Thus, it shows that despite there being institutionalized instruments for S&ID in the hemispheric dimension, it has not been producing dynamic results in terms of the continuous collaboration with countries in LA. The socio-economic asymmetries with countries in North America, with stronger national agendas for S&T, can be part of the explanation of this challenge.

Finally, IAI is an intergovernmental board created toward S&ID in 1992. Recently, IAI has been developing the IAI Science Technology & Policy (STeP) Fellowship through a Pilot Program 2020–2024 aiming at professional development on three initial pillars of science diplomacy, communication, and leadership. The program intends to create an Inter-American Network for shared capacity building and science-policy experiences among the fellows, host institutions, and IAI member country stakeholders. Created after the American Association for the Advancement of Science (AAAS) Science & Technology Policy Fellowship, an important Associate of the IAI, STeP aims at being the national level in LA, looking to expand new cohorts from the region (IAI, 2020).

**Figure 1:**
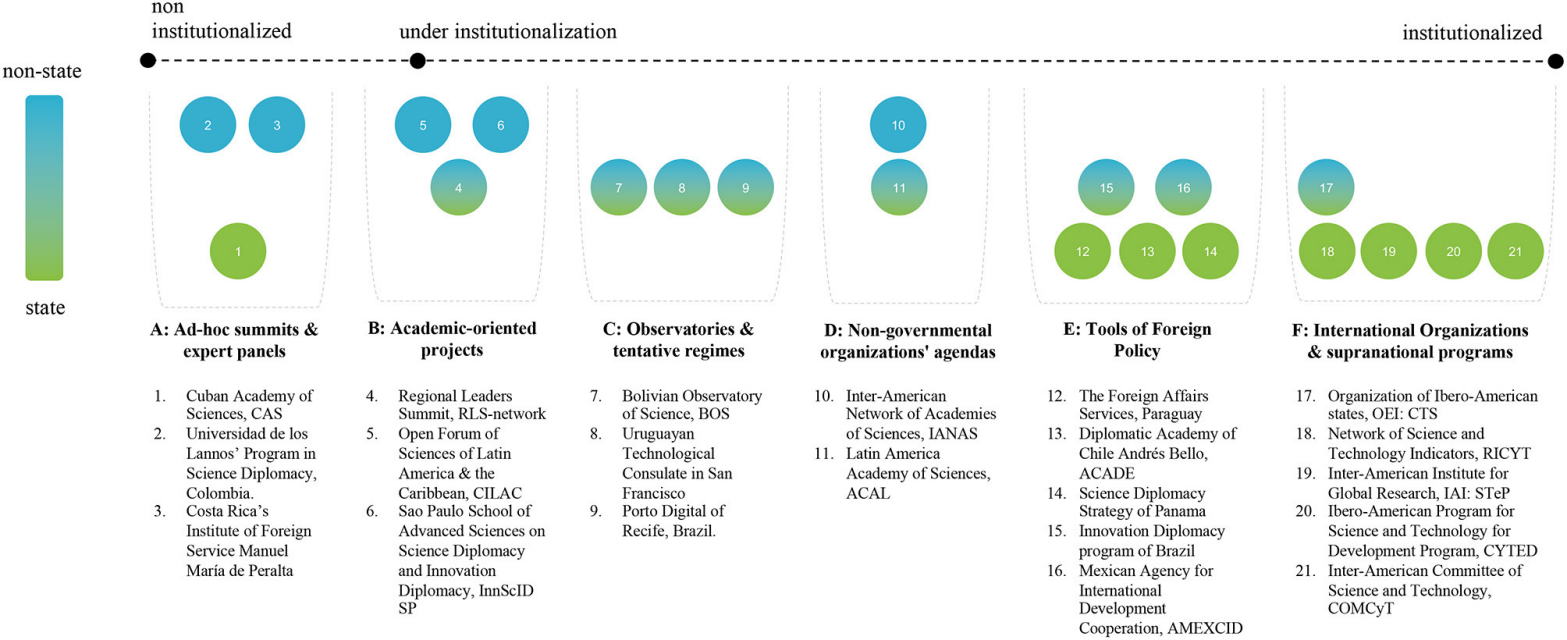
Case levels of institutionalization and level of state to non-state involvement.

A visual representation of previous experiences and policies provided as it follows (Figure 1). It illustrates how those initiatives can be localized in the proposed typology and in the framework of institutionalization level, but there is no statistical or precise quantitative inquiry.

### Limitations

Despite the presented policies and the selected cases bringing important initiatives of institutional building in S&ID in LA, the typology has some relevant limitations in terms of (1) lack or asymmetry of publications with qualified data about experiences in LA, since those are very recent initiatives, (2) information platforms/websites are still under construction, (3) initiatives of S&ID in LA continue to be analyzed mostly by researchers based in European countries, and (4) as result of all those problems, the proposed typology can only picture the characteristics of S&ID in LA with a few cases from countries with published reports/working papers.

## Discussion

### Can Foreign Policy Bridge the Scientific and Technological Gap?

What can be seen in most Latin American countries is the absence of S&T issues on the foreign policy agenda. In reality, the themes have always been treated separately and are still largely misunderstood.

In the early 1990's, with the depolarization of the international system, the optimistic expectation that globalization could bring benefits and increase the level of cooperation between countries prevailed. However, there is no denying that the global changes, encountered in the previous decades, have imposed challenges of the most different orders to scientific diplomacy in the last few decades, since climate change, the impacts of globalization and the innovation of bureaucratic instruments. All of these elements have forced constant changes on the part of researchers, in creating new instruments and new networks that guarantee the dissemination of scientific results and the creation of new collaborations capable of breaking continental boundaries.

This context of challenges was strongly impacted by the Covid-19 pandemic and accelerated the need for dialogue between countries and for advances in cooperation agreements between scientists. However, these initiatives often run into the limitations of the governments themselves.

The COVID-19 pandemic focused attention on this issue and on others involving cooperation between states and different actors. At the international level, one of the main challenges in times of crisis is to guarantee cooperation commitments between states and to strengthen the role of international organizations. The tendency of states, especially in the face of the health crisis confronting us, is to seek to protect the population, closing borders, and implementing protectionist policies.

What gives basis to the relevance of scientific diplomacy is the identification that several conflicts could be overcome through the cooperation of scientists from different nations. Furthermore, although there is still no structured system of articulation, as in developed countries, such as the United States and the United Kingdom–examples of countries that institutionalized this system and the implementation of S&T policies as part of the list of external actions–this demand has grown significantly.

A foreign policy that could more systematically incorporate issues of ST&I, allowing the expansion of cooperation agreements with other countries and the transfer of technology, as well as the sharing of innovations in different fields, would certainly have a significant impact on developing countries in LA. However, it is noted that Latin American countries lack investment in a better articulated agenda between scientific production and the foreign policy agenda that could reduce the gap with developed countries.

### Emergence of Subnational Actors

Over the last few decades, non-state actors have begun to occupy important spaces in terms of their contribution to various themes that directly affect states and individuals within the state borders. In several fields, as in ST&I, the interdependence of various actors to foster innovation and scientific advances in terms of financing, production of results, and scientific dissemination has become clear.

The institutional building in S&ID in LA is strongly related to the emergence of new subnational actors interested in the promotion of S&T, facing the decrease of dynamism and influence of traditional boards of foreign policy and of the National System of Innovation in the global arenas of ST&I. In LA, the twenty-first century is dealing with a political deficit with new approaches from the civic society, which has been led mainly by two relevant actors: the academic community based in universities and new international collaborations between NGOs, companies, and local governments.

As shown in the previous section, there are some international collaborations between local NGOs and local governments to foster S&ID that were implemented and pursued by academic-based actors (Gual-Soler, 2020). LA countries have been implementing relevant agendas of international collaboration with civic society, with an interesting level of success. Non-governmental actors and researchers have been collaborating in different initiatives promoted by the EU and other members of the business sector, which shows a strong subnational-based S&ID agenda.

Although universities, regional governments, and local municipalities do not have the legal prerogative to engage in international relations, regions have long realized that the international interface contributes to the improvement of public policies for local interests. Various cooperation agreements have been established between subnational entities creating results that are more visible to the citizens. This is true, especially when it comes to issues related to the field of innovation where the exchange of knowledge is directed to short- and mid-term policies, as well as the more effective involvement of other governmental and non-governmental actors.

In the field of S&ID, the demand from scientists and companies for greater interaction with government actors has been growing and not yet fully structured. There is still a lack of better understanding between the role of different levels of public administration in ST&I topics, both from the legal and political framework, i.e., which favors national states but also led to competition and not cooperation.

### Challenges of S&ID in LA

Competitiveness in innovation systems relies on the quality of the interactions between different actors. Therefore, the existence of different actors and multi-level governance/coordination capability between them within the initiatives, whether institutionalized or not, are crucial to the development efficient S&ID and competitive innovation. However, they must be able to build coordination capability between them on a long-term basis. In LA, among the intergovernmental endeavors, there are significant initiatives from the end of the 1980's and 1990's, focusing on science and technology in the region. Yet, many of them seem to lack a strong continuity.

S&ID systems in LA are quite heterogeneous throughout the region and not sufficiently structured, denoting a lack of strategy able to improve the quality of the interactions. It is possible to identify a higher level of interconnection between some regional initiatives; however, even though there have been regional forums and institutes, more executive initiatives are often local, lacking a connectedness between different LAC actors.

In this sense, there are specific initiatives in some countries, and collaborations also by individuals or between scientific groups of academic institutions, in general universities. However, in all Latin American countries, a public policy aimed at encouraging cooperation agreements and institutionalizing collaborations is necessary to improve the articulation between foreign policy and S&ID, in order to contribute to the development of countries.

EU is pushing S&ID to LA countries at various levels, developing projects and co-operated initiatives (Selleslaghs, 2017). The EU launched the National/Regional Innovation Strategies for Smart Specialization (RIS3) under the Cohesion Policy (2014–2020) instrument (European Commission, 2013). The Smart Specialization is a policy approach with a place-based dimension, aiming at exploiting advantages of proximity to promote economic growth and competitiveness. As reported by Sánchez (2018), RIS3 (i) intends to transform regional economies around new knowledge-based activity domains (ii) through an entrepreneurial discovery process between the public and private sectors (iii) to identify the most promising activities in which to specialize (iv) within a framework of multi-faced and multi-governance interactions.

For several years, projects from different countries in LA have received support from the European Commission, through calls for tenders for scientific and innovation projects, as well as support for networks that allow the exchange of knowledge between countries with a level of development. Other developed countries have also influenced S&ID in LA: the Spanish Foundation for Science and Technology (FECYT) and the American Association for the Advancement of Science (AAAS) have been developing many initiatives in the region^3^.

Although external pressure can accelerate the establishment of S&ID and create a more homogeneous global system, it can also create policies that do not meet local needs. The active participation of local scientists and policy makers is essential to the success of S&ID in developing quality interactions within the region. Even though there are important connections with the USA and the EU and countries specifically considered, there is a lack of integration between LA countries to build an organic and strong S&ID strategy.

The various experiences of regional integration in LA could have already advanced more robust cooperation projects in the area of scientific diplomacy and innovation, but this field was never given priority. The integration processes themselves would benefit, given that a broader integration has always been chosen, especially since the 1990's, involving government actors at the head of the negotiations, but also private actors. Integration processes, such as Mercosur, had the involvement of non-governmental actors who, on many occasions, have advanced cooperation even more than the governmental agreements themselves.

As a model, taking into account policies developed by the European Union, this could in fact contribute to advancing policies that allow a better articulation of the different actors, and between public policies and the foreign policy agenda. As has been shown, there is a multitude of actors that could benefit from an efficient articulation to be effective, and the region could learn from established and profitable initiatives.

## Conclusions

The paper centralizes the existence of multiple experiences of S&ID in LA, which deserves in-depth research and analysis. The main contribution here is to provide a simple typology regarding the varieties of S&ID initiatives and how its institutional building is being influenced by state and non-state actors, regionally and globally. The main finding is the necessity for better articulation between S&ID initiatives in and among LA countries, as well as a wider understanding of the dynamics of ST&I in GS countries, that brings challenges but also possibilities of open agendas. This articulation is key to integrate multiple subnational and non-state actors, addressing the improvement of the agendas of foreign policy toward more effective actions and instruments to foster the development of ST&I in the region. Consequently, it is necessary to understand S&ID as tools for social and economic development—and not an end in itself.

In this sense, the building of common ground, with shared values and goals, between those different actors and countries seems to be critical. From this understanding, it is possible to develop a strong and coherent framework outlining the short-, the medium-, and the long-term objectives and policies able to support the actions. This will influence greatly the *quality* of the interactions, orienting the results.

Many S&ID initiatives were identified in the LA region with different levels of institutionalization, involving different stakeholders and levels of governance. However, many of those initiatives seem to be fragmented and lacking steady continuity, denoting poor capacity to picture long-term agendas. This seems to be due to some limitations that are holding LA back, such as a weak S&T policy, low levels of independency of educational and scientific systems, and political instability.

The enormous potential for cooperation in S&ID between LA countries can clearly be seen, and this article can hopefully provide introductory tools for future work about this subject.

## Author Contributions

RS was the primary author and developed the paper proposal and it written, which includes conception, literature review, empirical design, data collection, data analysis, and the presentation of its main results and discussions. GF was responsible for data collection and analysis, writing, articulation of the parts, and the presentation of its main results and discussions. AO and JO participated actively in the written of results, discussion, and conclusion, as well as in the paper submission. All authors facilitated all levels of the documentary research and access to primary data (information about the FAPESP São Paulo Advanced School on Science and Innovation Diplomacy), as well as the construction of the database, case selection, the construction of the typology, and the discussion of results, also to providing assistance with data catalog, reviewing, and agreed with the submitted and reviewed version of the article.

## Conflict of Interest

The authors declare that the research was conducted in the absence of any commercial or financial relationships that could be construed as a potential conflict of interest.

## Footnotes

^1^As a matter of space and scope, since our work focuses on the presentation of an analytical typology, we will not go further on the reasons for the recent birth of S&ID in LA. For this discussion, see the concept of Latin American Thought in Science, Technology, and Development (PLACTED) in Sabato (2011).

^2^Two reports on the subject were published: one in 2018 by Centre of International Studies Gilberto Bosques, a technical support body to the Senate, and the other in 2020 by the SRE in partnership with the Barcelona Science and Technology Diplomacy Hub (Sci-Tech DiploHub).

^3^Spain launched the INTERCOONECTA, the Knowledge Transfer, Exchange and Management Plan for the Development of Spanish Cooperation in Latin America and the Caribbean, was created with the intention of integrating, coordinating, and reinforcing the action that Spanish Cooperation develops in terms of training and knowledge for more than 25 years. In 2017, within the framework of the INTERCOONECTA Plan for Spanish Cooperation, the Spanish Foundation for Science and Technology (FECYT) and the Spanish Agency for International Development Cooperation (AECID) organized the course “Scientific Diplomacy for managers in Latin America” aimed at managers of public organizations from Latin American countries and the Caribbean region. The course happened in Santa Cruz de la Sierra, Bolivia. FECYT was also present in the design of the Panamanian National Strategy on Science, Technology and Innovation, along with AAAS and UNESCO. More information about the Science Diplomacy course at https://www.fecyt.es/es/noticia/fecyt-organiza-un-curso-de-diplomacia-cientifica-en-latinoamerica-0. Accessed in November 2020. In addition, the SciTech DiploHub, a subnational S&ID initiative from Barcelona, created an important analysis note on S&ID in partnership with Institute Matías Romero, from the Mexican Government (Roig, 2020).
